# Impact of volume status on sarcopenia in non-dialysis chronic kidney disease patients

**DOI:** 10.1038/s41598-022-25135-z

**Published:** 2022-12-24

**Authors:** Seok Hui Kang, Jun Chul Kim, Ran-hui Cha, Miyeun Han, Won Suk An, Su Hyun Kim, Jun Young Do

**Affiliations:** 1 Division of Nephrology, Department of Internal Medicine, College of Medicine, Yeungnam University, Daegu, Republic of Korea; 2 Department of Internal Medicine, CHA Gumi Medical Center, CHA University, 12, Sinsi-ro 10-gil, Gumi, 39295 Republic of Korea; 3 Department of Internal Medicine, National Medical Center, 245, Eulji-ro, Jung-gu, Seoul, 04564 Republic of Korea; 4 Department of Internal Medicine, Hallym University Hangang Sacred Heart Hospital, 12, Beodeunaru-ro 7-gil, Yeongdeungpo-gu, Seoul, 07247 Republic of Korea; 5 Department of Internal Medicine, Dong-A University College of Medicine, 26, Daesingongwon-ro, Dongdaesin-dong 3-ga, Seo-gu, Busan, 49201 Republic of Korea; 6 Department of Internal Medicine, Chung-Ang University Hospital, Chung-Ang University College of Medicine, 102, Heukseok-ro, Dongjak-gu, Seoul, 06973 Republic of Korea

**Keywords:** Medical research, Nephrology

## Abstract

There were few data regarding the association of volume status with sarcopenia using muscle mass, strength, and physical performance in non-dialysis chronic kidney disease (ND-CKD) patients. We aimed to evaluate the association between volume status and sarcopenia in ND-CKD patients. Our retrospective study analyzed data from a previous study which included ND-CKD patients who had stable renal function. Our study used its baseline data alone. The edema index and muscle mass were measured using a multi-frequency bioimpedance analysis machine. The edema index was calculated using extracellular water/total body water ratio. The skeletal muscle index (SMI, kg/m^2^) was calculated using appendicular muscle mass per height squared. Handgrip strength (HGS, kg) was measured during the standing position in all patients. Dynamic gait speed (GS, m/s) was evaluated using 6-m walking speed. Patients with both low muscle mass (SMI < 7.0 kg/m^2^ for men and < 5.7 kg/m^2^ for women using bioimpedance analysis) and low HGS (< 28 kg for men and < 18 kg for women) or low GS (< 1.0 m/s) were classified as having sarcopenia. The patients (n = 147) were divided into tertiles based on the edema index level. The mean edema index in the low, middle, and high tertiles was 0.377 ± 0.006, 0.390 ± 0.003, and 0.402 ± 0.006, respectively. The edema index was significantly correlated with SMI, HGS, and GS (*r* = *− *0.343 for SMI, *− *0.492 for HGS, and *− *0.331 for GS; *P* < 0.001 for three indicators). The SMI, HGS, and GS values were 8.1 ± 1.0 kg/m^2^, 33.0 ± 9.4 kg, and 1.2 ± 0.2 m/s in the low tertile,7.8 ± 1.2 kg/m^2^, 30.0 ± 7.5 kg, and 1.0 ± 0.3 m/s in the middle tertile, and 7.2 ± 1.4 kg/m^2^, 23.7 ± 7.4 kg, and 1.0 ± 0.3 m/s in the high tertile, respectively. Univariate analyses revealed that SMI was lower in patients in the high tertile than in those in the low tertile. HGS was lowest in high tertile, and GS was greatest in the low tertile. The high tertile for predicting sarcopenia had an odds ratio of 6.03 (95% CI, 1.78–20.37; *P* = 0.004) compared to low or middle tertiles. The results of multivariate analyses were similar to those of the univariate analyses. The subgroup analyses showed that statistical significance was greater in < 65 years and men than ≥ 65 years and women. The present study showed that the edema index is inversely associated with sarcopenia, muscle mass index, strength, and physical performance in ND-CKD patients. However, considering the limitations of our study such as its small sample size, this association was not strong. Further studies that include volume-independent measurements, data on physical activity and diet, and a larger number of patients are warranted to overcome these limitations.

## Introduction

Chronic kidney disease (CKD) is an increasing health problem worldwide. The prevalence of CKD is approximately 11.1% in Korea and 14.9% in USA^1,2^. Two main functions of the kidneys are the removal of uremic toxins and the control of volume and electrolytes. Non-dialysis CKD (ND-CKD) patients are frequently prone to develop volume overload. Volume overload in CKD patients is associated with various complications, including increased blood pressure, congestive heart failure, and rapid progression of CKD, resulting in a high risk of cardiovascular disease or mortality. Recent data also indicate metabolic complications in addition to mechanical complications by simple volume overload.

Early screening and improved care for CKD patients improve long-term survival in CKD patients. The risk of long-term complications due to CKD may be increased, and clinicians should pay particular attention to identifying these problems. Sarcopenia is an important long-term complication in CKD patients, which is more likely with increasing age and improved long-term survival. Sarcopenia in CKD patients is classically developed through protein-energy wasting due to the muscular effects of inflammation and insulin resistance^3^. However, extramuscular factors, such as neurohormonal effects, also lead to sarcopenia in CKD patients without muscle catabolism. Previous studies involving dialysis-dependent CKD (DD-CKD) patients showed that sarcopenia is developed by increased muscle catabolism and decreased muscle function through volume overloading or uremia^4,5^. Hypervolemic patients, such as those undergoing dialysis, were associated with increased extracellular water compared to normovolemic patients^6^. Extracellular fluid overload leads to endotoxin translocation, decreased splanchnic blood flow, and increased sodium consumption, which results in systemic inflammation^7^. These findings indicate that hypervolemia may be associated with sarcopenia through systemic inflammation. However, there were few data regarding the association of volume status with sarcopenia using muscle mass, strength, and physical performance in ND-CKD patients. We aimed to evaluate the association between volume status and sarcopenia in ND-CKD patients.

## Methods

### Study population

Our retrospective study analyzed data from a previous study^8^. Briefly, the RECOVERY study was a randomized controlled trial that evaluated the effect of AST-120 on sarcopenia in ND-CKD patients who had stable renal function with a serum creatinine level between 2.0 and 5.0 mg/dL or an estimated glomerular filtration rate (eGFR) between 15 and 60 mL/min/1.73m^2^ for 3 months. The study was longitudinal in design over 48 weeks, but our study used its baseline data alone. A total of 150 participants were randomized, of which three were excluded due to missing data. Therefore, 147 ND-CKD patients were finally included. This study received ethical approval from the Institutional Review Board of a local medical center (approval No: 2018-09-006). The written informed consent was obtained from all subjects and/or their legal guardians. The study was conducted ethically in accordance with the World Medical Association Declaration of Helsinki. Finally, the patients were divided into tertiles based on the edema index level as follows: low, middle, and high.

### Clinical variables

We collected baseline data including age, sex, comorbidities, body mass index (kg/m^2^), eGFR (mL/min/1.73m^2^), and high sensitivity C-reactive protein (hs-CRP, mg/dL), tumor necrosis factor-α (TNF-α, pg/mL), interleukin-6 (pg/mL), calcium (mg/dL), phosphorus (mg/dL), sodium (mmol/L), potassium (mmol/L), albumin (g/dL), alkaline phosphatase (IU/L), and intact parathyroid hormone (i-PTH, ng/mL) levels. Comorbidity was evaluated using Charlson’s comorbidity index (CCI) score with a previously described method^9^. The eGFR was calculated using the CKD-Epidemiology Collaboration equation with a previously described method^10^. CKD stages were also defined according to the Kidney Disease: Improving Global Outcomes guidelines^11^.

### Assessment of volume status, sarcopenia, and health-related quality of life scales

The edema index and muscle mass were measured using a multi-frequency bioimpedance analysis machine (InBody S10, Seoul, Korea). These measurements were measured using previous protocols^4,8^. Briefly, each subject was clothed with a lightweight gown and emptied their bladder. Measurements were performed after 5 min of rest in the supine position. Eight electrodes were placed—two for each foot and two for each hand—with the patient in the supine position. The machine measured extracellular water and total body water content, and the edema index was calculated using extracellular water/total body water ratio^4^. The skeletal muscle index (SMI, kg/m^2^) was calculated using appendicular muscle mass per height squared.

Handgrip strength (HGS, kg) was measured during standing position in all patients. Each patient performed three trials with their dominant hand using a digital dynamometer (Takei 5401; Takei Scientific Instruments Co., Ltd, Niigata, Japan). The maximum strength measured over the three trials was recorded. Dynamic gait speed (GS, m/s) was evaluated using 6 m walking speed. The participants started walking 2 m prior to the measurement point (acceleration zone) and ended 2 m after the measurement point (deceleration zone). GS measurements were performed twice, and the mean value was recorded.

In our study, sarcopenia was defined according to the Asian Working Group for Sarcopenia^12^. Patients with both low muscle mass (SMI < 7.0 kg/m^2^ for men and < 5.7 kg/m^2^ for women using bioimpedance analysis) and low HGS (< 28 kg for men and < 18 kg for women) or low GS (< 1.0 m/s) were classified as having sarcopenia^12^.

Health related quality of life (HR-QoL) was assessed using the Kidney Disease Quality of Life (KDQOL)-SF™ 1.3 Korean version^13^. KDQOL-SF™ 1.3 includes the SF-36 scale (36 items) and kidney disease-specific scale (11 items). The SF-36 includes eight domains: physical functioning (PF), role limitations due to physical health problems (RP), body pain, general health, vitality, social functioning, role limitations due to emotional problems (RE), mental health, and overall health rating (OHR). A total score of 0–100 is calculated for each domain. A low score indicates a low quality of life. These scales are used to calculate the physical component scale (PCS) and mental component scale (MCS)^14,15^. The kidney disease-specific 11 items include symptom/problems, kidney disease effects, kidney disease burden, work status, cognitive function, quality of social interaction, sexual function, sleep, social support, patient satisfaction, and dialysis staff encouragement^13–15^. Among these, patient satisfaction and dialysis staff encouragement were not obtained as ND-CKD patients were included.

### Statistical analysis

The data were analyzed using IBM SPSS Statistics version 25 (SPSS Inc., Chicago, IL, USA). All statistical tests were two-tailed. Categorical variables were expressed as counts (percentages). All continuous variables were expressed as mean and standard deviations, except the results from analysis of covariance. Variables of analysis of covariance for multivariate analysis were expressed as mean and standard error. For continuous variables, means were compared using one-way analysis of variance, followed by a Bonferroni post-hoc comparison and analysis of covariance for multivariate analysis. The correlation between two continuous variables was assessed using Pearson’s or partial correlation analyses. In addition, we performed linear regression analyses using the SMI, HGS, or GS as dependent variables. Logistic regression analysis was performed to evaluate the independent predictor of sarcopenia. Covariates were selected as variables with statistically significant differences among three tertiles. Multivariate analysis was adjusted for age, sex, CCI score, eGFR, and calcium, phosphorus, albumin, and i-PTH levels. The area under the receiver operating characteristic curve (AUROC) was used to calculate the probability of predicting sarcopenia, sensitivity, and specificity. MedCalc version 11.6.1.0 (MedCalc, Mariakerke, Belgium) was used for AUROC. The level of statistical significance was set at *P* < 0.05.

## Results

### Participants’ clinical characteristics

The mean edema index in the low, middle, and high tertiles was 0.377 ± 0.006 (interval, 0.362–0.384), 0.390 ± 0.003 (interval, 0.385–0.395), and 0.402 ± 0.006 (interval, 0.396–0.417), respectively. The patients in the high tertile were older than those in the other tertiles, and the proportion of male sex was lowest in the high tertile (Table 1). The CCI score was greatest in the high tertile, and eGFR, calcium, and albumin levels were lower in the high tertile than in the low tertile. The phosphorus and i-PTH levels were greater in the high tertile than in the low tertile.

**Table 1:** Participants’ clinical characteristics.

	Total (n = 147)	Low tertile (n = 49)	Middle tertile (n = 49)	High tertile (n = 49)	*P* -value
Age (years)	65.0 ± 10.8	60.0 ± 9.6	65.0 ± 11.2^+^	70.1 ± 9.2^+#^	< 0.001
Sex (men)	96 (65.3%)	37 (75.5%)	34 (69.4%)	25 (51.0%)	0.030
CCI score	3.9 ± 1.9	2.8 ± 1.7	3.9 ± 1.7^+^	4.9 ± 1.7^+#^	< 0.001
eGFR (mL/min/1.73m^2^)	33.8 ± 12.5	38.2 ± 11.8	33.7 ± 12.0	29.5 ± 12.4^#^	0.002
Body mass index (kg/m^2^)	25.3 ± 3.0	25.7 ± 2.5	25.7 ± 3.3	24.5 ± 3.2	0.076
hs-CRP (mg/dL)	0.46 ± 1.23	0.41 ± 1.18	0.57 ± 1.24	0.41 ± 1.29	0.824
TNF-α (pg/mL)	1.90 ± 1.58	2.06 ± 2.19	1.93 ± 1.53	1.72 ± 0.67	0.585
Interleukin-6 (pg/mL)	2.39 ± 1.76	2.35 ± 2.09	2.33 ± 1.21	2.50 ± 1.87	0.882
Calcium (mg/dL)	9.1 ± 0.6	9.3 ± 0.5	9.1 ± 0.6	8.9 ± 0.5^#^	0.011
Phosphorus (mg/dL)	3.5 ± 0.6	3.4 ± 0.5	3.4 ± 0.6	3.7 ± 0.6^#^	0.011
Sodium (mmol/L)	140 ± 2	141 ± 2	140 ± 2	141 ± 2	0.158
Potassium (mmol/L)	4.8 ± 0.6	4.8 ± 0.6	4.9 ± 0.6	4.8 ± 0.5	0.578
Serum albumin (g/dL)	4.3 ± 0.4	4.4 ± 0.3	4.3 ± 0.3	4.2 ± 0.4^#^	0.017
Alkaline phosphatase (IU/L)	88 ± 60.2	76 ± 20	99 ± 96	89 ± 35	0.168
i-PTH (ng/mL)	104.5 ± 81.4	66.7 ± 35.5	87.0 ± 74.4	104.5 ± 81.4^#^	0.022

Data are expressed as the mean ± standard deviation and as numbers (percentages) for categorical variables. *P-*values were tested using one-way analysis of variance, followed by Bonferroni’s post-hoc comparison for continuous variables and Pearson’s χ^2^ or Fisher’s exact tests for categorical variables.

CCI, Charlson comorbidity index; eGFR, estimated glomerular filtration rate; hs-CRP, high-sensitivity C-reactive protein; TNF, tumor necrosis factor; i-PTH, intact parathyroid hormone.

^+^*P* < 0.05, compared to the low tertile, and ^#^*P* < 0.05, compared to the middle tertile.

### Association between edema index and sarcopenia components

**Figure 1:**
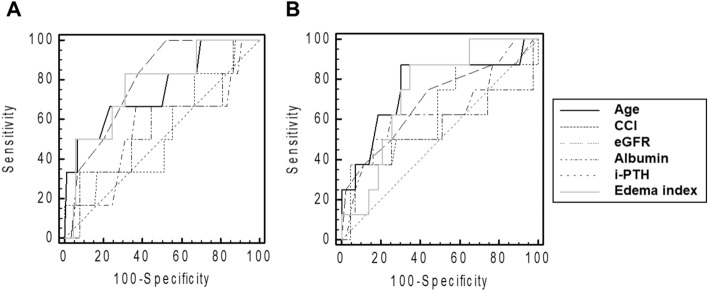
Receiver operating characteristic curve of the edema index used to predict sarcopenia in men (**A**) and in women (**B**). CCI, Charlson comorbidity index; eGFR, estimated glomerular filtration rate; i-PTH, intact parathyroid hormone.

Pearson’s correlation coefficients with edema index were − 0.343 for SMI, − 0.492 for HGS, and − 0.331 for GS, respectively (*P* < 0.001 for three indicators). Partial correlation coefficients with the edema index were − 0.191 for SMI, − 0.337 for HGS, and − 0.278 for GS, respectively (*P* = 0.024 for SMI, *P* < 0.001 for HGS, and *P* = 0.001 for GS). Univariate and multivariate linear regression analysis showed that the edema index as a continuous variable was inversely associated with the SMI, HGS, and GS (Table S1). The AUROCs of edema index for sarcopenia were 0.77 (95% confidence interval [CI], 0.67–0.85; *P* = 0.010, Fig. 1A) in men and 0.74 (95% CI, 0.60–0.85; *P* = 0.004, Fig. 1B) in women. The sensitivity and specificity for predicting sarcopenia were 83.3% and 69.8%, respectively, in men and 87.5% and 65.1%, respectively, in women.

For men, the AUROCs were 0.75 (95% CI, 0.65–0.83), 0.79 (95% CI, 0.70–0.87), 0.54 (95% CI, 0.43–0.64), 0.55 (95% CI, 0.45–0.65), and 0.56 (95% CI, 0.46–0.66) for age, CCI score, eGFR, albumin, and i-PTH, respectively. For women, the AUROCs were 0.76 (95% CI, 0.62–0.87), 0.69 (95% CI, 0.54–0.81), 0.64 (95% CI, 0.49–0.77), 0.65 (95% CI, 0.50–0.77), and 0.54 (95% CI, 0.40–0.68) for age, CCI score, eGFR, albumin, and i-PTH, respectively. The AUROCs of the edema index for sarcopenia were greatest among the six variables, except for the CCI score in men and age in women. Furthermore, we calculated age-adjusted AUROCs using the kernel-based estimation of the covariate-adjusted ROC curve. The age-adjusted AUROCs were 0.69 in men and 0.61 in women, and the values decreased compared to those without adjustment.

**Figure 2:**
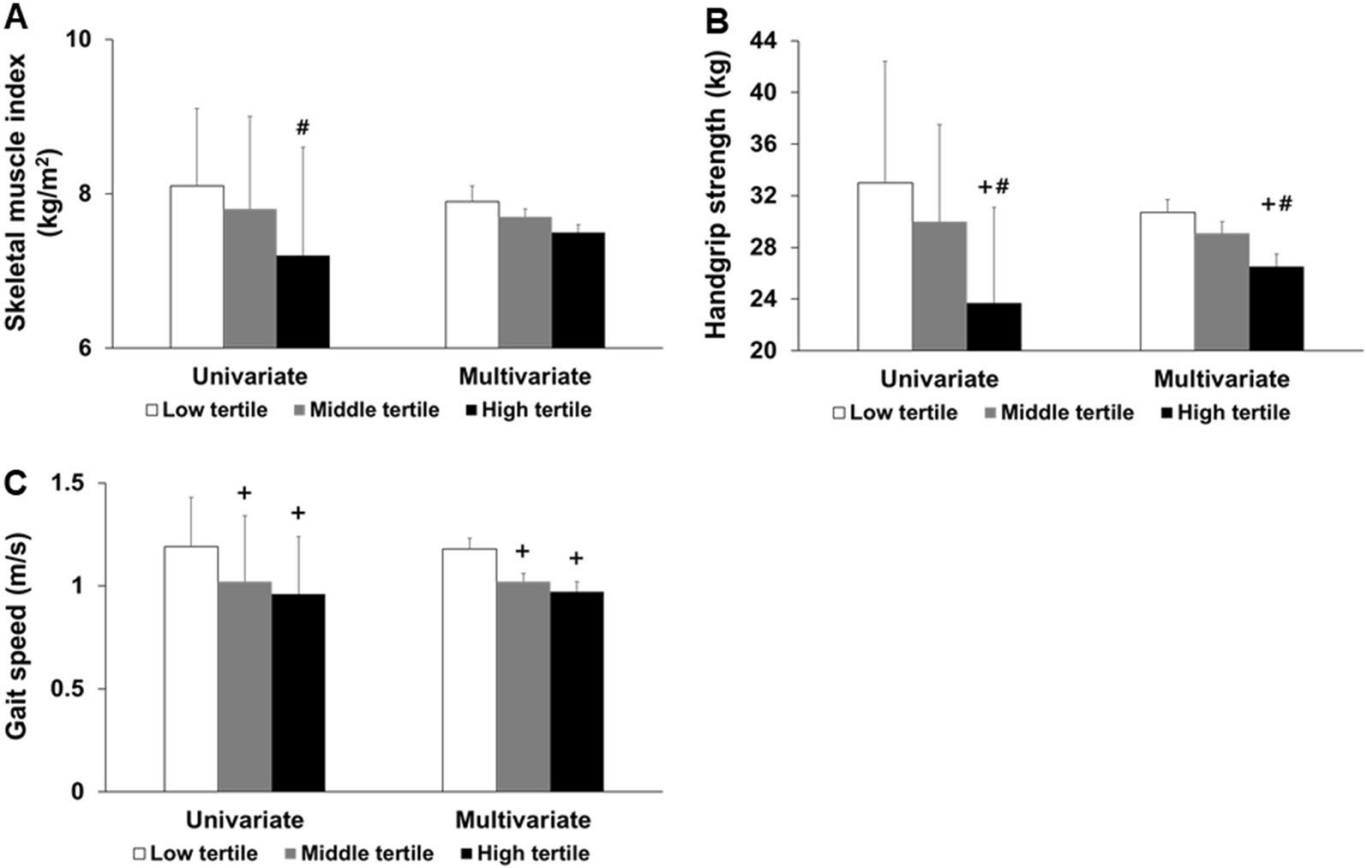
Comparisons of the skeletal muscle index (**A**), handgrip strength (**B**), and gait speed (**C**) according to tertiles of the edema index. Data were expressed as the mean (standard deviation) in univariate analyses and as the mean (standard error) in multivariate analyses. Univariate analysis was conducted using one-way analysis of variance followed by Bonferroni’s post-hoc comparison, and multivariate analysis was conducted using analysis of covariance after adjusting for age, sex, Charlson comorbidity index, estimated glomerular filtration rate, calcium, phosphorus, albumin, and intact parathyroid hormone.^+^*P* < 0.05, compared to the low tertile, and ^#^*P* < 0.05, compared to the middle tertile.

Univariate analyses revealed that SMI was lower in patients in the high tertile than in those in the low tertile (Fig. 2). HGS was lowest in high tertile, and GS was greatest in the low tertile. Univariate logistic regression analysis showed that the high tertile for predicting sarcopenia had an odds ratio of 6.03 (*P* = 0.004) compared to low or middle tertiles (Table 2). The results of multivariate analyses were similar to those of the univariate analyses. Furthermore, we performed logistic regression analyses using categorized variables to avoid the effect of collinearity and these were similar with those from continuous variables (Table S2).

**Table 2:** Logistic regression analysis for sarcopenia according to variables.

	Univariate		Multivariate	
	Odds ratio (95% CI)	*P*-value	Odds ratio (95% CI)	*P*-value
Age (ref: < 65 years)	5.91 (1.27–27.43)	0.023	4.32 (0.83–22.58)	0.083
Sex (ref: men)	2.76 (0.90–8.44)	0.075	3.69 (0.86–15.77)	0.079
CCI score (per 1 score increase)	1.20 (0.86–1.67)	0.285	1.24 (0.83–1.87)	0.298
eGFR (per 1 ml/min/1.73 m^2^ increase)	0.96 (0.92–1.01)	0.124	0.98 (0.92–1.04)	0.516
Calcium (per 1 mg/dL increase)	0.88 (0.33–2.37)	0.802	1.30 (0.30–5.55)	0.725
Phosphorus (per 1 mg/dL increase)	1.25 (0.51–3.10)	0.625	0.49 (0.14–1.74)	0.270
Albumin (per 1 g/dL increase)	1.65 (0.32–8.38)	0.547	3.30 (0.44–24.61)	0.245
i-PTH (per 1 ng/mL increase)	1.00 (1.00–1.01)	0.317	1.00 (0.99–1.01)	0.959
Edema index (ref: low or middle tertile)	6.03 (1.78–20.37)	0.004	4.28 (1.01–18.17)	0.048

Multivariate analysis was adjusted for age, sex, CCI score, eGFR, calcium, phosphorus, albumin, i-PTH, and edema index.

CI, confidence interval; CCI, Charlson comorbidity index; eGFR, estimated glomerular filtration rate; i-PTH, intact parathyroid hormone.

### Subgroup analyses according to age, sex, or CKD stages

We divided the patients into two age groups. For those aged < 65 years, three indicators in univariate and multivariate showed significant association with the edema index (Table 3). No statistical significances were obtained in those aged ≥ 65 years on multivariate analyses. On analyses by sex, HGS and GS were associated with edema index on multivariate analysis in men, but not in women. The numbers of patients according to the CKD stage were 36 (24.5%), 50 (34%), 53 (36.1%), and 8 (5.4%) for G3a, G3b, G4, and G5, respectively. The proportions of patients with sarcopenia according to CKD stages were 2 (5.6%), 4 (8.0%), 4 (7.5%), and 4 (50%) for G3a, G3b, G4, and G5, respectively (*P* = 0.001). Pearson’s correlation coefficients with the edema index are shown in Table S3. The proportion of patients with sarcopenia increased as the CKD stage advanced. Subgroup analyses according to CKD stages showed that the associations between edema index and sarcopenia-related indicators were generally inverse; however, the association with SMI was relatively weaker than that with HGS or GS. Statistical significance for correlations was not obtained in G5; however, the trends were similar to those from other groups.

**Table 3:** Correlation between the edema index and sarcopenia-related indicators according age or sex.

	Age				Sex			
	< 65 years (n = 68)		≥ 65 years (n = 79)		Men (n = 96)		Women (n = 51)	
	*r*	*P*-value	*r*	*P*-value	*r*	*P*-value	*r*	*P*-value
**Pearson’s correlation**								
Skeletal muscle index	− 0.482	< 0.001	− 0.168	0.139	− 0.266	0.009	− 0.282	0.045
Handgrip strength	− 0.556	< 0.001	− 0.360	0.001	− 0.537	< 0.001	− 0.277	0.049
Gait speed	− 0.226	0.028	− 0.253	0.025	− 0.384	< 0.001	− 0.130	0.362
**Partial correlation**								
Skeletal muscle index	− 0.395	0.002	− 0.040	0.741	− 0.202	0.058	− 0.224	0.144
Handgrip strength	− 0.508	< 0.001	− 0.203	0.088	− 0.412	< 0.001	− 0.162	0.294
Gait speed	− 0.309	0.015	− 0.216	0.069	− 0.307	0.003	0.049	0.750

Partial correlation was adjusted for age, sex, Charlson comorbidity index score, estimated glomerular filtration rate, and calcium, phosphorus, albumin, and intact parathyroid hormone levels.

### Association between edema index and HR-QoL scales

Among SF-36 scales, PF, RP, RE, OHR, PCS, and MCS were associated with tertiles of edema index, and these scales were greatest in the low tertile (Table S4). Among kidney disease-specific scales, symptom/problems, work status, cognitive function, quality of social interaction, sexual function, social support, and OHR were associated with tertiles of edema index, and all scales excluding symptom/problems were greatest in the low tertile.

## Discussion

In our study, associations between the edema index and sarcopenia or sarcopenia components were mainly evaluated. The edema index was inversely correlated with SMI, HGS, and GS. The tertiles of the edema index were inversely associated with HGS and GS on univariate and multivariate analyses. The association between the edema index and SMI was weak compared to HGS or GS. Baseline characteristics, such as age or sex, were different among the tertiles of the edema index, and we additionally performed subgroup analyses using these variables. The results showed that statistical significance was greater in < 65 years and men than ≥ 65 years and women. We additionally evaluated the association between the tertiles of the edema index and HR-QoL scales and showed favorable outcomes in the low tertile group.

Previous studies evaluated the association between volume status and malnutrition in CKD patients^16–20^. Some studies involving DD-CKD patients evaluated volume status and nutritional status, which were evaluated using a serologic marker, such as N-terminal fragment of B-type natriuretic peptide or serum albumin, or bioimpedance^16–18^. These studies showed a positive association between volume status and malnutrition and suggested that the possible interaction between the two variables is inflammation. Two studies including ND-CKD patients showed similar results to those using DD-CKD patients^19,20^.

Previous studies that evaluated association between volume status and malnutrition have some limitations. First, cross-talk between volume status and malnutrition were identified as inflammation in DD-CKD, but studies using ND-CKD did not fully evaluate inflammatory markers. Second, outcome measurements were limited to simple muscle mass measurements using bioimpedance, intracellular water, or serum albumin. Therefore, our study included ND-CKD patients alone and evaluated sarcopenia as a hard outcome; sarcopenia components including muscle mass index, strength, and physical performance; and inflammatory indicators including hs-CRP, TNF-α, and interleukin-6. Our study revealed that volume status is associated with sarcopenia or sarcopenia components and there is no significant difference in inflammatory indicators and serum albumin levels among the three tertiles. ND-CKD patients were less uremic and had less volume overload compared to DD-CKD patients. Therefore, the pathophysiology between volume overload and malnutrition in ND-CKD patients may differ from that in DD-CKD patients. Our study revealed that the cross-talk between volume status and sarcopenia or sarcopenia components may be associated with other pathways rather than inflammation and malnutrition.

One possible mediator would be derived from cardiac sarcopenia/cachexia associated with mainly volume overload. Heart failure patients are prone to develop sarcopenia, and the pathophysiology includes neurohormonal activation, peripheral ischemia, reduced oxygen delivery and physical activity^21^. Although the relationship among muscle mass, strength, and physical performance was complex, a decrease in muscle volume per se would be more closely associated with catabolic status, such as inflammation, in CKD patients. Inflammation by volume overload would be more likely to develop in DD-CKD patients than ND-CKD patients. Strength and physical performance may be influenced by both muscle volume and neural factors^22,23^. Our study showed that volume overload is more closely associated with HGS and GS than SMI. Although mediators between volume and sarcopenia in ND-CKD patients is beyond our study’s field, factors other rather than inflammation may influence the development of sarcopenia, especially functional factors, by volume overload.

Results from subgroup analyses are other important issue. The association between volume status and sarcopenia was greater in younger patients and in the male sex than in the elderly and female sex. Elderly CKD patients have decreased anabolic hormone levels compared to those of the general population^24^. A normal or accelerating aging process also occurs in elderly CKD patients. Conversely, women are more prone than men to the effect of sex hormones. A combination of other factors, such as the aging process and the effects of sex or anabolic hormones, may attenuate the impact of volume on the development of sarcopenia.

Two recent guidelines from the European and Asian Working groups recommend that sarcopenia should be defined as both low muscle quality and low muscle quantity^12,25^. The guidelines from the Asian Working group defines sarcopenia as low skeletal muscle mass plus loss of muscle strength and/or reduced physical performance^12^. Sarcopenia in our study was defined according to this concept. Previous studies have defined sarcopenia as any one of the following three factors: low muscle mass, low muscle strength, or low physical performance. However, these can be strictly defined as dynaphenia (low muscle strength or low physical performance alone) or presarcopenia (low muscle mass alone), and recent guidelines are against the use of these concepts due to insufficient evidence regarding the association between these definitions and prognosis^12,25^.

Our results may provide some information for ND-CKD patients with volume overload. Some special instruments are needed to evaluate sarcopenia and it is difficult to screen or evaluate sarcopenia using these instruments for all patients. Considering the positive association between volume overload and sarcopenia, ND-CKD patients with volume overloading may be ideal candidates for deciding whether further assessments for a final diagnosis of sarcopenia are needed. In addition, the effectiveness of treatment in CKD patients with sarcopenia is not high^26^. It is important to perform pre-emptive intervention and early identification for patients at risk of sarcopenia before clinically evident symptoms or signs appear. These findings reveal that identification of volume overloading may be helpful in the early diagnosis of sarcopenia in ND-CKD patients through pre-emptive evaluation, which would be helpful in attenuating the progress of sarcopenia or improving the patient’s responsiveness to treatment, which will enable the clinician to reverse sarcopenia through pre-emptive interventions.

Our study has some limitations. First, our study was retrospective and a post-hoc analysis of baseline data from a previous study. Second, it has as methodological limitation. Muscle mass and volume status were measured using bioimpedance. However, the bioimpedance method is not the gold standard despite its accuracy in general population. Volume overload patients have biased results compared to normohydrated participants^27^. Third, our study included patients with a broad range of eGFR and did not include data for physical activity and diet. Physical activity and diet are important confounding factors regarding the development of sarcopenia. Although our data did not include physical activity, there was no significant difference in the metabolic equivalent for task among the three tertiles (data were not shown). Fourth, baseline characteristics were significantly different among the three tertiles. Factors such as age, sex, and CCI score influence sarcopenia and its components. However, we tried to attenuate these confounding factors using multivariate or subgroup analyses.

In conclusion, the present study showed that the edema index is inversely associated with sarcopenia, muscle mass index, strength, and physical performance in ND-CKD patients. Our data may allow clinical practitioners to evaluate sarcopenia in ND-CKD patients with volume overload and provide them with various interventions for preventing or treating sarcopenia. However, considering the limitations of our study such as its small sample size, the association was not strong. Further studies that include volume-independent measurements, data for physical activity and diet, and a larger number of patients are warranted to overcome these limitations.

## Supplementary Information

Supplementary Table S1.

Supplementary Table S2.

Supplementary Table S3.

Supplementary Table S4.

## Data Availability

All data relevant to the study are included in the article or uploaded as supplementary information.
