# Supplementary material for: Impact of volume status on sarcopenia in non-dialysis chronic kidney disease patients
Source: Sci Rep. 2022 Dec 24;12:22289. doi: 10.1038/s41598-022-25135-z (PMC9789973; doi:10.1038/s41598-022-25135-z)
Supplement: Supplementary file 1 — Supplementary Table S1. [file 41598_2022_25135_MOESM1_ESM.docx]

**Table S1. Linear regression analysis of sarcopenia-related components according to variables**

|  | **Univariate** | | **Multivariate** | |
| --- | --- | --- | --- | --- |
|  | St-β (SE) | *P*-value | St-β (SE) | *P*-value |
| Dependent variable: SMI |  |  |  |  |
| Age (per 1 year increase) | –0.14 (0.01) | 0.095 | –0.13 (0.10) | 0.118 |
| Sex (ref: men) | –0.57 (0.18) | <0.001 | –0.50 (0.19) | <0.001 |
| CCI score (per 1 score increase) | –0.09 (0.05) | 0.284 | 0.02 (0.06) | 0.803 |
| eGFR (per 1 ml/min/1.73 m^2^ increase) | 0.29 (0.01) | <0.001 | 0.10 (0.01) | 0.231 |
| Calcium (per 1 mg/dL increase) | –0.01 (0.18) | 0.867 | –0.05 (0.19) | 0.570 |
| Phosphorus (per 1 mg/dL increase) | –0.25 (0.16) | 0.002 | 0.00 (0.16) | 0.990 |
| Albumin (per 1 g/dL increase) | –0.07 (0.29) | 0.383 | –0.16 (0.28) | 0.048 |
| i-PTH (per 1 ng/mL increase) | –0.22 (0.00) | 0.008 | –0.06 (0.00) | 0.492 |
| Edema index (per 0.001 unit increase) | –0.34 (0.01) | <0.001 | –0.20 (0.01) | 0.024 |
| Dependent variable: HGS |  |  |  |  |
| Age (per 1 year increase) | –0.22 (0.07) | 0.006 | –0.18 (0.06) | 0.006 |
| Sex (ref: men) | –0.68 (1.13) | <0.001 | –0.60 (1.14) | <0.001 |
| CCI score (per 1 score increase) | –0.17 (0.39) | 0.039 | 0.02 (0.32) | 0.730 |
| eGFR (per 1 ml/min/1.73 m^2^ increase) | 0.25 (0.06) | 0.002 | –0.06 (0.05) | 0.404 |
| Calcium (per 1 mg/dL increase) | 0.12 (1.32) | 0.137 | 0.07 (1.10) | 0.307 |
| Phosphorus (per 1 mg/dL increase) | –0.33 (1.14) | <0.001 | –0.09 (0.92) | 0.151 |
| Albumin (per 1 g/dL increase) | 0.05 (2.10) | 0.549 | –0.10 (1.63) | 0.113 |
| i-PTH (per 1 ng/mL increase) | –0.22 (0.01) | 0.007 | –0.01 (0.01) | 0.867 |
| Edema index (per 0.001 unit increase) | –0.49 (0.06) | <0.001 | –0.30 (0.05) | <0.001 |
| Dependent variable: GS |  |  |  |  |
| Age (per 1 year increase) | –0.20 (0.00) | 0.017 | –0.07 (0.00) | 0.483 |
| Sex (ref: men) | –0.12 (0.05) | 0.158 | –0.06 (0.06) | 0.470 |
| CCI score (per 1 score increase) | –0.22 (0.01) | 0.008 | –0.04 (0.02) | 0.668 |
| eGFR (per 1 ml/min/1.73 m^2^ increase) | –0.07 (0.00) | 0.370 | –0.29 (0.00) | 0.004 |
| Calcium (per 1 mg/dL increase) | 0.05 (0.04) | 0.543 | –0.08 (0.05) | 0.455 |
| Phosphorus (per 1 mg/dL increase) | –0.08 (0.04) | 0.327 | –0.08 (0.05) | 0.411 |
| Albumin (per 1 g/dL increase) | 0.08 (0.07) | 0.305 | –0.07 (0.08) | 0.463 |
| i-PTH (per 1 ng/mL increase) | –0.03 (0.00) | 0.742 | –0.05 (0.00) | 0.599 |
| Edema index (per 0.001 unit increase) | –0.33 (0.00) | <0.001 | –0.35 (0.00) | 0.001 |

Multivariate analysis was adjusted for age, sex, CCI score, eGFR, calcium, phosphorus, albumin, i-PTH, and edema index.

Abbreviations: St-β, standardized beta; SE, standardized error; SMI, skeletal muscle index; CCI, Charlson comorbidity index; eGFR, estimated glomerular filtration rate; i-PTH, intact parathyroid hormone; HGS, handgrip strength; GS, gait speed.
