# Supplementary material for: Impact of volume status on sarcopenia in non-dialysis chronic kidney disease patients
Source: Sci Rep. 2022 Dec 24;12:22289. doi: 10.1038/s41598-022-25135-z (PMC9789973; doi:10.1038/s41598-022-25135-z)
Supplement: Supplementary file 2 — Supplementary Table S2. [file 41598_2022_25135_MOESM2_ESM.doc]

**Table S2. Logistic regression analysis for sarcopenia according to variables**

|  | **Univariate** | | **Multivariate** | |
| --- | --- | --- | --- | --- |
|  | **Odds ratio (95% CI)** | ***P*-value** | **Odds ratio (95% CI)** | ***P*-value** |
| Age (ref: < 65 years) | 2.23 (0.42–11.90) | 0.346 | 1.76 (0.26–11.79) | 0.561 |
| Sex (ref: men) | 1.42 (0.31–6.62) | 0.652 | 2.24 (0.26–19.50) | 0.465 |
| CCI score (ref: < 4) | 2.30 (0.43–12.24) | 0.330 | 0.92 (0.10–8.59) | 0.939 |
| eGFR (ref: < 33.6ml/min/1.73 m^2^) | 1.33 (0.29–6.17) | 0.713 | 2.87 (0.42–19.68) | 0.284 |
| Calcium (ref: < 9.1 mg/dL) | 1.19 (0.26–5.52) | 0.823 | 4.04 (0.56–28.99) | 0.166 |
| Phosphorus (ref: < 3.5 mg/dL) | 0.73 (0.16–3.38) | 0.686 | 0.26 (0.03–2.09) | 0.206 |
| Albumin (ref: < 4.3 g/dL) | 0.25 (0.05–1.35) | 0.108 | 0.11 (0.01–0.94) | 0.044 |
| i-PTH (ref: <68.1 ng/mL) | 0.75 (0.16–3.47) | 0.713 | 0.55 (0.09–3.32) | 0.513 |
| Edema index (per increase 1 tertile) | 4.29 (1.07–17.15) | 0.040 | 6.90 (1.02–46.52) | 0.047 |

Multivariate analysis was adjusted for age, sex, CCI score, eGFR, calcium, phosphorus, albumin, i-PTH, and edema index.

Abbreviations: CI, confidence interval; CCI, Charlson comorbidity index; eGFR, estimated glomerular filtration rate; i-PTH, intact parathyroid hormone.
