# Supplementary material for: Impact of volume status on sarcopenia in non-dialysis chronic kidney disease patients
Source: Sci Rep. 2022 Dec 24;12:22289. doi: 10.1038/s41598-022-25135-z (PMC9789973; doi:10.1038/s41598-022-25135-z)
Supplement: Supplementary file 3 — Supplementary Table S3. [file 41598_2022_25135_MOESM3_ESM.doc]

**Table S3. Correlation between the edema index and sarcopenia-related indicators according chronic kidney disease stage**

|  | **G3a (n = 36)** | | **G3b (n = 50)** | | **G4 (n = 53)** | | **G5 (n = 8)** | |
| --- | --- | --- | --- | --- | --- | --- | --- | --- |
|  | ***r*** | ***P*-value** | ***r*** | ***P*-value** | ***r*** | ***P*-value** | ***r*** | ***P*-value** |
| Pearson’s correlation |  |  |  |  |  |  |  |  |
| Skeletal muscle index | –0.515 | 0.001 | –0.125 | 0.388 | –0.221 | 0.112 | –0.358 | 0.384 |
| Handgrip strength | –0.511 | 0.001 | –0.405 | 0.004 | –0.406 | 0.003 | –0.550 | 0.158 |
| Gait speed | –0.488 | 0.003 | –0.247 | 0.084 | –0.372 | 0.006 | –0.694 | 0.056 |
