# Supplementary material for: Impact of volume status on sarcopenia in non-dialysis chronic kidney disease patients
Source: Sci Rep. 2022 Dec 24;12:22289. doi: 10.1038/s41598-022-25135-z (PMC9789973; doi:10.1038/s41598-022-25135-z)
Supplement: Supplementary file 4 — Supplementary Table S4. [file 41598_2022_25135_MOESM4_ESM.docx]

**Table S4. Comparison of quality of life scale scores by tertiles of edema index**

| **Short form-36 scale** |  | **Low tertile** | **Middle tertile** | **High tertile** | ***P*-value** |
| --- | --- | --- | --- | --- | --- |
| Physical functioning |  | 89.4 ± 12.4 | 75.9 ± 20.7^#^ | 66.8 ± 24.7^#^ | <0.001 |
| Role limitations due to physical health problems |  | 81.6 ± 30.5 | 60.7 ± 39.9^#^ | 62.2 ± 42.7^#^ | 0.012 |
| Bodily pain |  | 80.7 ± 23.6 | 76..5 ± 23.0 | 71.4 ± 23.2 | 0.145 |
| General health |  | 46.9 ± 16.6 | 43.1 ± 15.1 | 39.4 ± 17.5 | 0.079 |
| Vitality |  | 53.0 ± 19.0 | 48.3 ± 17.4 | 47.4 ± 15.1 | 0.236 |
| Social functioning |  | 88.8 ± 16.4 | 81.1 ± 25.7 | 81.6 ± 25.1 | 0.182 |
| Role limitations due to emotional problems |  | 87.1 ± 28.7 | 60.5 ± 43.9^#^ | 66.0 ± 45.9^#^ | 0.003 |
| Mental health |  | 66.3 ± 13.4 | 62.0 ± 16.6 | 60.5 ± 16.7 | 0.166 |
| Overall health rating |  | 32.1 ± 17.7 | 24.0 ± 15.3^#^ | 20.9 ± 17.2^#^ | 0.004 |
| Physical component scale |  | 74.7 ± 16.5 | 64.0 ± 19.5^#^ | 60.0 ± 21.3^#^ | 0.001 |
| Mental component scale |  | 79.8 ± 14.8 | 63.0 ± 21.6^#^ | 63.9 ± 20.5^#^ | 0.001 |
| **Kidney disease-specific scale** |  |  |  |  |  |
| Symptom/problems |  | 88.4 ± 11..7 | 82.3 ± 14.1 | 80.6 ± 17.0^#^ | 0.010 |
| Kidney disease effects |  | 88.0 ± 14.1 | 84.5 ± 14.4 | 80.4 ± 18.6 | 0.061 |
| Kidney disease burden |  | 66.8 ± 22.4 | 62.9 ± 22.8 | 56.4 ± 26.8 | 0.098 |
| Work status |  | 84.0 ± 29.7 | 47.7 ± 42.2^#^ | 29.7 ± 34.3^#^ | <0.001 |
| Cognitive function |  | 90.2 ± 11.1 | 82.0 ± 17.5^#^ | 82.4 ± 18.0^#^ | 0.018 |
| Quality of social interaction |  | 84.6 ± 13.8 | 69.3 ± 18.0^#^ | 72.2 ± 18.6^#^ | <0.001 |
| Sexual function |  | 89.8 ± 14.7 | 56.8 ± 31.8^#^ | 73.4 ± 34.4 | 0.001 |
| Sleep |  | 69.1 ± 13.8 | 66.0 ± 16.0 | 67.1 ± 21.3 | 0.665 |
| Social support |  | 77.5 ± 16.2 | 63.6 ± 23.0^#^ | 62.2 ± 22.0^#^ | <0.001 |
| Overall health rating |  | 67.8 ± 18.4 | 56.5 ± 13.5^#^ | 52.7 ± 17.4^#^ | <0.001 |

Comparisons were tested by one-way analysis of variance, followed by a Bonferroni’s post-hoc comparison. ^#^*P* < 0.05 versus the low tertile group.
